# The Effect of Consumer-based Activity Tracker Intervention on Physical Activity among Recent Retirees—An RCT Study

**DOI:** 10.1249/MSS.0000000000002627

**Published:** 2021-02-08

**Authors:** TUIJA LESKINEN, KRISTIN SUORSA, MIIKA TUOMINEN, ANNA PULAKKA, JAANA PENTTI, ELIISA LÖYTTYNIEMI, ILKKA HEINONEN, JUSSI VAHTERA, SARI STENHOLM

**Affiliations:** 1 Department of Public Health, University of Turku and Turku University Hospital, Turku, FINLAND; 2 Centre for Population Health Research, University of Turku and Turku University Hospital, Turku, FINLAND; 3 Finnish Institute for Health and Welfare, Helsinki, FINLAND; 4 Clinicum, Faculty of Medicine, University of Helsinki, Helsinki, FINLAND; 5 Department of Biostatistics, University of Turku and Turku University Hospital, Turku, FINLAND; 6 Turku PET Centre, and Department of Clinical Physiology and Nuclear Medicine, University of Turku, Turku, FINLAND; 7 Rydberg Laboratory of Applied Sciences, Department of Environmental and Biosciences, University of Halmstad, Halmstad, SWEDEN

**Keywords:** PHYSICAL ACTIVITY, RCT, WEARABLE TECHNOLOGY, RETIREMENT, OLDER ADULTS

## Abstract

**Purpose:**

The randomized controlled trial REACT (NCT03320746) examined the effect of a 12-month consumer-based activity tracker intervention on accelerometer-measured physical activity among recent retirees.

**Methods:**

Altogether 231 recently retired Finnish adults (age, 65.2 ± 1.1 yr, mean ± SD; 83% women) were randomized to intervention and control groups. Intervention participants were requested to wear a commercial wrist-worn activity tracker (Polar Loop 2; Polar, Kempele, Finland) for 12 months, to try to reach the daily activity goals shown on the tracker display, and to upload their activity data to a Web-based program every week. The control group received no intervention. Accelerometer-based outcome measurements of daily total, light physical activity (LPA), and moderate to vigorous (MVPA) physical activity were conducted at baseline and at 3-, 6-, and 12-month time points. Hierarchical linear mixed models were used to examine the differences between the groups over time. All analyses were performed by intention-to-treat principle and adjusted for wake wear time.

**Results:**

The use of a commercial activity tracker did not increase daily total activity, LPA, or MVPA over the 12-months period when compared with nonuser controls (group–time interaction, *P* = 0.39, 0.23, and 0.77, respectively). There was an increase in LPA over the first 6 months in both the intervention (26 min·d^−1^, 95% confidence interval [CI] = 13 to 39) and the control (14 min·d^−1^, 95% CI = 1 to 27) groups, but the difference between the groups was not significant (12 min·d^−1^, 95% CI = −6 to 30). In both groups, LPA decreased from 6 to 12 months.

**Conclusion:**

The 12-month use of a commercial activity tracker does not appear to elicit significant changes in the daily total activity among a general population sample of recent retirees, thus highlighting the need to explore other alternatives to increase physical activity in this target group.

## Abstract 2

Supplemental digital content is available in the text.

The number of retired adults is increasing worldwide. Strategies to support retirees to be more physically active in their daily lives are important for the maintenance of health and mobility with advancing age (1). Among other life transitions and events, retirement can be regarded as an opportunity for individuals to change their physical activity behavior along with the increased time available and restructured leisure activities, which may facilitate physical activity (2–4). Observational follow-up studies have shown that self-reported leisure-time physical activity increases during the retirement transition (5–8), but so does self-reported sitting time (8–11). More recent accelerometer-based studies have found that women decrease their daily total activity (12) whereas men remain highly sedentary (13) after retirement transition. Therefore, retirement appears to be an important and perhaps the most optimal time point to intervene, either to maintain or to promote physical activity at older age (11,14,15). However, according to the literature, there are only few studies on physical activity interventions targeted to recently retired adults (16–19); thus, the potential of this period is not fully explored.

Traditional physical activity interventions, such as group or individual counseling and training sessions, are reported to be effective in improving physical activity levels up to 12 months in older adults, but the evidence relies mostly on subjectively measured outcomes (16,20). Unlike face-to-face interventions requiring facilities and time, various e-health interventions, such as Web- or mobile-based interventions, are more scalable and accessible to promote physical activity and also feasible as technology can be harnessed to deliver behavioral change techniques (21). Overall, e-health interventions have shown to be acceptable for older population and effective in increasing physical activity up to 6 months in adults over 60 yr of age (18,21–23).

Previous multicomponent e-health interventions have used wearable devices, such as pedometers and accelerometers, as a supplement to other intervention components or to measure the outcomes (24–26). Commercially available activity or fitness trackers (e.g., Fitbit) are increasingly used as the core instruments of technology-aided interventions (27) as they offer evidence-based self-management strategies, such as goal setting and feedback (28). However, previous consumer-based activity tracker interventions with accelerometer-measured physical activity as an outcome have been of short duration (3 to 6 months) (21,27), and only a few studies have been conducted among adults over 60 yr of age (24). A Web-based intervention (Philips DirectLife), including an accelerometer-based activity monitor, a personal Web site, and an e-coach, increased moderate to vigorous physical activity (MVPA) by 11 min·d^−1^ in 12 wk among inactive 60 to 70 yr old adults (29). A Fitbit-based intervention increased weekly MVPA by 62 min in 16 wk among inactive postmenopausal women (30). Also, the use of the Jawbone Up24 wearable activity monitor and app, including weekly phone calls during the intervention, increased daily activity in 12 wk among sedentary 60 yr old adults (31). Furthermore, there are other multicomponent physical activity interventions that have found that using commercial activity trackers among older adults benefits a physical activity intervention (32,33). However, the independent and long-term (>6 months) effect of commercial activity tracker on daily total physical activity among older adults has not yet been studied.

The primary aim of the Enhancing Physical Activity and Healthy Aging among Recent Retirees (REACT) trial was to evaluate the effect of a 12-month consumer-based activity tracker intervention, as compared with controls not using any activity trackers, on accelerometer-measured daily total physical activity, light physical activity (LPA), and MVPA in recently retired Finnish adults. The hypothesis was that daily total activity increases to a significantly greater extent within the intervention group than within the control group.

## METHODS

### Participants

#### Recruitment of the participants

The target group for the REACT trial included Finnish public sector employees whose estimated statutory retirement dates were between January 2016 and April 2019 and who lived in the region of Southwest Finland in 2017. The information on the estimated individual statutory retirement dates was obtained from the pension insurance institute for the municipal sector in Finland (Keva). The number of eligible individuals was 1475 (1166 women and 309 men), and they were first contacted with a letter mailed to their home addresses in January 2018. The letter included detailed information of the REACT trial, the inclusion criteria, and a link to a short Web-based questionnaire, which aimed at collecting data on the demographic characteristics, current health status, actual dates of retirement, and e-mail addresses for those who got interested. The enrollment continued to March 2018.

The inclusion criteria for the REACT trial were the following: self-reported actual date of retirement between January 2016 and December 2018, self-reported ability to walk 500 m without interruption, no current postoperative state, no known surgery within the next 6 months, no malign cancer or recent myocardial infraction, basic knowledge on how to use a computer, and Internet access at home. In Finland, it is possible to continue working after statutory retirement, and an irregular or part time job was not used as an exclusion criterion.

**FIGURE 1:**
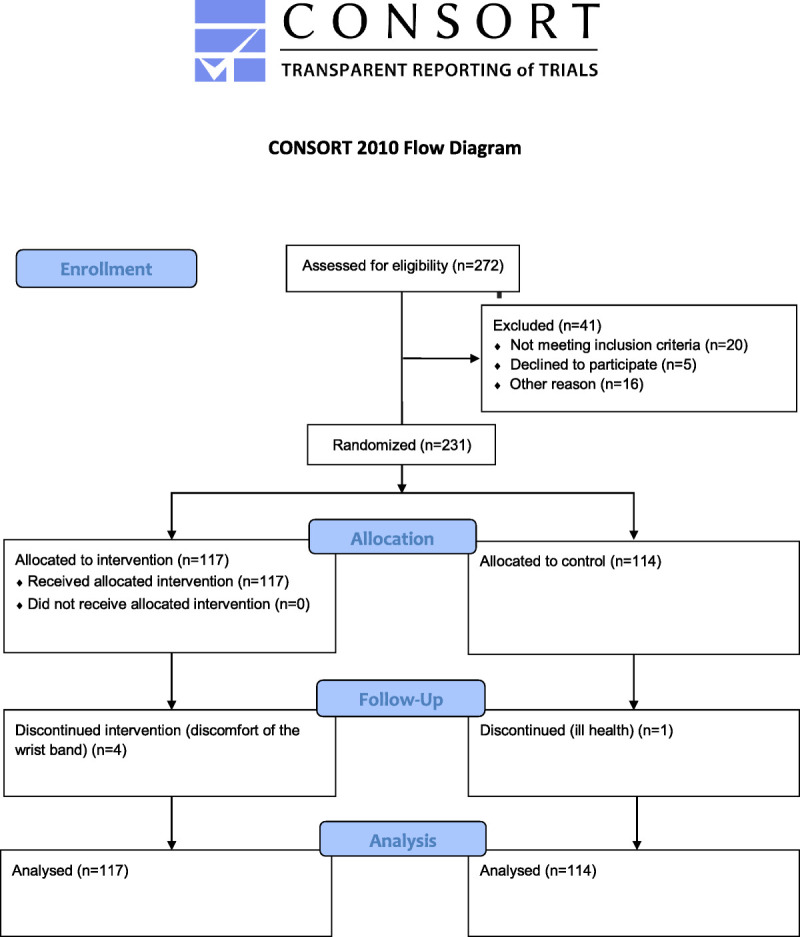
Flow diagram of the REACT trial.

Overall, 272 individuals (18.4% of those eligible) expressed their interest to take part in the study. The respondents were more frequently women (82% vs 78%) and highly educated (37% vs 20%) compared with nonrespondents (*n* = 1203). There was no difference in the mean ± SD age (65.1 ± 1.2 vs 64.9 ± 1.3 yr). Of the respondents, 252 individuals were invited to the study as we did not include individuals who had retired before January 2016 (*n* = 12) or individuals who reported they will retire after the year of 2018 (*n* = 8). Finally, 231 recent retirees were able to participate and 21 were not. Figure 1 presents the flow diagram of participation. Baseline measurements were conducted on average 1.2 ± 0.6 yr after the actual date of retirement.

#### Randomization

After the baseline measurements, all participants were randomized into two groups with an allocation ratio of 1:1. A statistician not involved in the running of the REACT trial prepared the randomization lists stratified by gender and using a random permuted block method with SAS software. The randomization slips were sealed in opaque envelopes in a numerical order. The envelopes were opened for each participant in the order of their clinical visits by a researcher. After the randomization, the initialized activity trackers, along with detailed instructions on how to use them and the manufacturer’s Web-based program/application, were mailed to the intervention group members. The control group members were informed of their allocation by e-mail.

#### Power calculation

The power calculation was based on a previous finding of an increase of 11% ± 31% in the wrist-worn accelerometer activity counts among 60 to 70 yr old adults after using a commercial activity monitor and a Web-based physical activity program for 3 months (29). We aimed to detect a 12% unit difference in daily total activity between the intervention and the control groups (assuming a 12% mean change in the intervention group and no mean change in the control group) after 12 months. Based on a power of 0.80 and a two-sided alpha of 0.05, the required sample size for the REACT trial was 214 participants. By taking into account a dropout rate of 10%, 240 participants was the goal set for the recruitment.

#### Ethics

The study follows the guidelines of good scientific practice set by the National Advisory Board on Research Ethics in Finland and the Declaration of Helsinki. The REACT trial has been approved by the ethics committee of the Hospital District of Southwest Finland (107/1801/2017), and its ClinicalTrials.gov registration number is NCT03320746. All participants were informed of the study protocol and voluntariness before they expressed their willingness to participate and gave a signed, informed consent.

### Intervention

The participants randomized to the intervention group were requested to wear a commercial wrist-worn activity tracker (Polar Loop 2; Polar, Kempele, Finland) on their nondominant wrist every day and night for 12 months. The Polar Loop 2 activity tracker included multiple behavior change techniques, such as goal setting, self-monitoring, and feedback, which are evidence-based self-management strategies (28) and comparable with those in other commercial activity trackers (34). As several features of the Polar Loop 2 were built around the daily activity goal, the daily activity goal attainment was chosen as the behavioral target for the intervention in order to maintain concordance between the goals and the means of the intervention. No further counseling or guidance on how to achieve the daily activity goal was given to the participants.

The participants were instructed to pursue the daily activity goal, initially set at stage 1 as per the goals set by the tracker manufacturer. The preset stages in activity goals were built around user’s typical daily activities, and they were also sensitive to the user’s gender and age. Because the Polar Loop 2 is tracking activity with a built-in accelerometer, various kinds of activities contribute to the achieving of the daily activity goal: activities at higher intensities helped to reach the daily goal faster than activities at lower intensities. The achievement of 100% of the daily activity goal at stage 1 corresponded, for example, to 57 min of jogging, or 2 h 11 min of walking, or 7 h 20 min of household chores, or a combination of activities at different intensities. At stage 1, the amount of daily activity necessary to achieve the goal exceeded the recommendation of weekly 150 min of MVPA (35).

The activity tracker enabled the user to monitor the real-time achievement of the activity goal and, e.g., the accumulation of daily steps. Based on the accumulated daily activity, the tracker provided feedback and displayed practical guidance on how to reach the remaining part of the daily goal, e.g., “jog for 20 minutes” or “walk for 50 minutes.” Upon 100% fulfillment of the daily goal, the tracker congratulated the user. Participants who frequently achieved or exceeded 100% of their daily activity goals at stage 1 were suggested by the researcher, via e-mail or SMS, to move on to stage 2 (activity goal comparable with ~3 h·d^−1^ of walking) and ultimately stage 3 (activity goal comparable with ~3.5 h·d^−1^ of walking). In some cases, the users changed the stage by themselves. The tracker also gave an inactivity alert by vibrating after 55 min of a nonmovement period, coupled with a prompt “it’s time to move” shown on the display (see Table, Supplemental Digital Content 1, Intervention content described in terms of behavior change techniques for details of the intervention content, http://links.lww.com/MSS/C265).

A researcher created personal accounts for the participants in Polar’s Web-based program (Polar Flow), to which the participants were requested to upload their activity tracker data at least once a week. The participants had unrestricted access to their personal Polar Flow accounts using a computer or mobile phone app. The uploading of the data from the tracker with a computer required the opening of Polar Flow in a Web browser. Polar Flow displayed overviews and summaries of the activity data on a daily, weekly, and monthly basis. Polar Flow also provided feedback on the attainment of the daily activity goal, and if the tracker had been worn sufficiently, a detailed feedback on the health benefits of accumulated activity, sedentary time, and sleep on daily, weekly, and monthly levels (see Figure, Supplemental Digital Content 2, Polar Flow diary view, http://links.lww.com/MSS/C266). The information from Polar Flow enabled researchers to follow the monthly use of the trackers, and if any lack of data was observed, the participant was contacted and requested to synchronize activity data from the tracker to Polar Flow. The activity data obtained from Polar Flow were also used to follow the achievement of the daily activity goal and to evaluate the dose of activity.

The control group members were requested to abstain from the use of any type of activity trackers during the 12-month follow-up period, and they were informed that they will receive Polar Loop 2 activity trackers and guidance for using them after the follow-up.

### Measurements

#### Accelerometer-measured physical activity

Wake-time physical activity was measured with wrist-worn triaxial ActiGraph wGT3X-BT accelerometers at 80-Hz sample frequency. The participants were requested to wear the accelerometers on their nondominant wrists. Accelerometer measurements lasting eight days and seven nights were conducted at baseline and at 3-, 6-, and 12-month follow-up time points for all the study participants. During the measurement weeks, the participants were also requested to fill in daily logs to indicate in-bed and out-bed times. Accelerometer data were collected between February 2018 and January 2020. Participants were treated in five waves, with the follow-up starting at spring season (44%), autumn season (25%), and winter season (31%). The intervention participants wore both devices, the Polar Loop 2 and the ActiGraph accelerometer, on their nondominant wrists during the follow-up measurement weeks. The ActiGraph accelerometer was always worn more distally and the Polar activity tracker more proximally on the wrist. Only if a participant reported discomfort while wearing of these two devices on the same wrist, the participant was instructed to wear the activity tracker on his/her dominant wrist and keep using the ActiGraph accelerometer on the nondominant wrist.

The accelerometer data were analyzed according to a prespecified data reduction and analysis plan and blinded for the allocation of the participants. Data from the ActiGraph accelerometers were downloaded using the ActiLife software (ActiGraph, Pensacola, FL) and processed using the open source R-package GGIR (R Foundation for Statistical Computing, Vienna, Austria, (https://cran.r-project.org/). The GGIR package has been developed for processing raw acceleration data from wrist-worn accelerometers into physical activity and sleep variables (36). Non–wear time was detected as a part of the GGIR processing (37), and sleep time was estimated based on the method of van Hees and coauthors (38), using both in-bed and out-bed times in the daily logs and algorithm of the GGIR package. Sleep and non–wear time were then excluded from the analysis. Wake-time total physical activity was determined as the sum of the time spent in LPA and MVPA, using the previously proposed threshold values: ≥30.0 m*g* for LPA and ≥100.6 m*g* for MVPA (39,40). At each time point, accelerometer measurements with at least four valid days with a minimum of 10 h of wear time during waking hours were considered valid, resulting to the exclusion of one follow-up measurement from two of the participants. The average number of valid days was 7.5 (range, 4 to 9). The mean values of wake time total activity for each 24 h of the measurement days were calculated, including only hours with at least 59 min of accelerometer recording (~88% of all measurement hours), and then averaged for each hour of the day across all valid days to illustrate daily physical activity patterns.

#### Background characteristics

The main background characteristics (date of birth, gender, and occupation) of the participants were derived from the pension institute’s register. Occupational status was categorized on the basis of the International Standard Classification of Occupations (ISCO) (41) into three groups: “high,” including managers and professionals (ISCO classes 1–2); “intermediate,” including associate professionals (ISCO classes 3–4); and “low,” including manual and service workers (ISCO classes 5–9) by the last known occupation preceding the retirement. Body mass index was calculated from the measured height and weight during the baseline clinical measurements. Other baseline characteristics were assessed by a Web-based questionnaire. Data on chronic conditions (none, 1, or >1) were based on a question, “Has your doctor ever told that you have or have had …” and the following diseases were taken into account: angina pectoris, myocardial infarction, stroke, claudication, osteoarthritis, osteoporosis, sciatica, fibromyalgia, rheumatoid arthritis, depression or other mental illness, and diabetes. Limitations in walking a distance of 2 km (yes vs no) were evaluated with the 36-Item Short Form Health Survey (42). Self-reported physical activity was assessed with a question concerning the average weekly duration and intensity of leisure-time physical activity during the past 3 months, and it was expressed as weekly metabolic equivalent (MET) hours.

### Statistical Analysis

Baseline characteristics of the study participants are presented as numbers and percentages for the categorical variables and as mean and SD values for the continuous variables. All analyses were performed by intention-to-treat principle so that all randomized participants were included in the analyses. Hierarchical linear mixed models were used to examine the differences in total physical activity (primary outcome), including LPA and MVPA, and wake wear time between the groups. The model included intervention group as a between-factor, time as a within-factor, and the group–time interaction. For the secondary analyses, we stratified the study participants into tertiles according to the daily total physical activity at baseline: low (47 to 229 min), middle (229 to 318 min), and high (318 to 546 min). We then examined the changes in physical activity by the baseline physical activity tertiles using hierarchical linear mixed models. Because the group–time interaction was significant for wake wear time (*P* = 0.04, partly due to differences in sleep time between the groups), all analyses were adjusted for wake wear time of the accelerometer. All results are shown as mean estimates and 95% confidence intervals (CI). The SAS Software 9.4 was used for the statistical analyses (SAS Institute Inc., Cary, NC).

## RESULTS

The age of the 231 randomized participants was 65.2 ± 1.1 yr (range, 61.8 to 67.6 yr); 83% were women, 35% had normal weight, and 38% had high occupational status. The baseline characteristics for the intervention and control group participants are shown in Table 1. The mean monthly active time as derived from the activity trackers remained relatively stable across the 12-month intervention (see Table, Supplemental Digital Content 3, Active time per each intervention month from the Polar Loop 2 activity tracker data, http://links.lww.com/MSS/C267).

**TABLE 1:** Baseline characteristics of the participants in the intervention and control groups.

Characteristics	Intervention (*n* = 117)	Control (*n* = 114)
	*n* (%)	*n* (%)
Age, mean ± SD, yr	65.2 (1.0)	65.2 (1.1)
Gender		
Women	96 (82.0)	95 (83.3)
Men	21 (18.0)	19 (16.7)
Occupational status		
High	47 (40.2)	41 (36.0)
Intermediate	35 (29.9)	28 (24.5)
Low	35 (29.9)	45 (39.5)
Body mass index		
Under/normal weight	38 (32.5)	43 (37.7)
Overweight	44 (37.6)	45 (39.5)
Obese	35 (29.9)	26 (22.8)
Chronic conditions		
0	36 (30.8)	27 (23.9)
1	47 (40.2)	45 (39.8)
>1	34 (29.1)	41 (36.3)
Limitations in walking 2 km*^a^*		
No	109 (93.2)	106 (93.8)
Yes	8 (6.8)	7 (6.2)
Self-reported physical activity (mean ± SD), MET·h·wk^−1^	29.9 (21.8)	29.1 (21.8)
Years from retirement transition (mean ± SD)	1.2 (0.6)	1.1 (0.5)

**^*a*^**From the 36-Item Short Form Health Survey.

**FIGURE 2:**
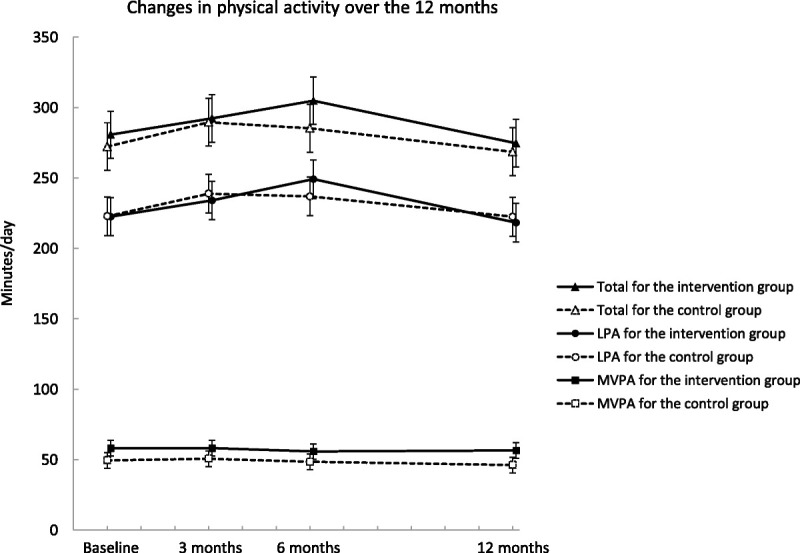
The change in total physical activity, LPA, and MVPA during the follow-up for the intervention (*solid line*) and control (*dotted line*) groups. Results are expressed as mean values and 95% CI based on mixed models.

**FIGURE 3:**
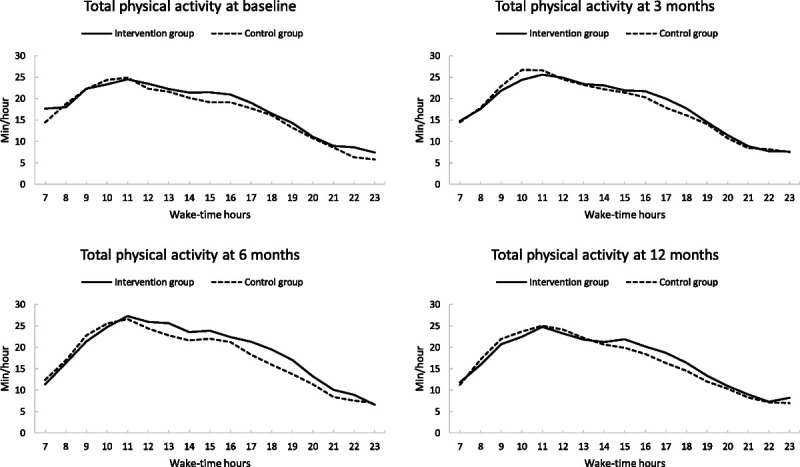
Daily profiles of the mean values of hourly total physical activity at each follow-up time point for the intervention (*solid line*) and control (*dotted line*) groups. Average values are based on mixed models.

There was no significant intervention effect in the accelerometer-measured daily total activity (group–time interaction *P* = 0.39), LPA (*P* = 0.23), or MVPA (*P* = 0.77) over the 12 months (Table 2). The intervention group members increased their daily total activity by 24 min·d^−1^ (95% CI = 10 to 38) and LPA by 26 min·d^−1^ (95% CI = 14 to 39) over the first 6 months, but the difference between the changes of the groups was not significant (11 min·d^−1^, 95% CI = −9 to 31 for total, and 12 min·d^−1^, 95% CI = −6 to 30 for LPA). Total and LPA levels returned close to the baseline levels in both groups at 12 months (Fig. 2). No differences in the change in MVPA over time were observed between the groups. Daily profiles of the mean values of hourly activity showed that the intervention group had slightly more of total activity from midday to late evening hours at 6 months, but no notable differences were observed in other time points (Fig. 3).

**TABLE 2:** Intention-to-treat analysis of the change in accelerometer-measured daily total physical activity, LPA, MVPA, and wake wear time from baseline to 3-, 6-, and 12-month time points.

		Intervention				Control			*P* Values		
Outcome Measure	*n*	Mean	95% CI		*n*	Mean	95% CI		Time Effect		Group–Time Interaction
Total physical activity, min·d^−1^										
Baseline	117	280.8	264.1	297.6	114	272.4	255.4	289.4		
Change from baseline to 3 months	113	11.5	−2.7	25.7	114	17.3	3.2	31.4		
Change from baseline to 6 months	113	23.9	9.7	38.2	112	13.0	−1.2	27.2		
Change from baseline to 12 months	113	−6.0	−20.1	8.1	112	−3.7	−17.9	10.5	<0.0001		0.39
LPA, min·d^−1^										
Baseline		222.7	209.0	236.3		222.9	209.1	236.7		
Change from baseline to 3 months		11.4	−1.3	24.0		16.1	3.5	28.7		
Change from baseline to 6 months		26.2	13.5	38.9		14.1	1.4	26.8		
Change from baseline to 12 months		−4.3	−16.9	8.2		−0.4	−13.0	12.3	<0.0001		0.23
MVPA, min·d^−1^										
Baseline		58.2	52.7	63.6		49.5	44.0	55.0		
Change from baseline to 3 months		0.03	−4.3	4.3		1.2	−3.1	5.5		
Change from baseline to 6 months		−2.3	−6.6	2.0		−1.1	−5.4	3.2		
Change from baseline to 12 months		−1.7	−6.0	2.6		−3.3	−7.6	1.0	0.16		0.77
Wake wear time, min·d^−1^										
Baseline		930.4	920.9	939.8		932.3	922.7	941.8		
Change from baseline to 3 months		14.6	6.1	23.2		2.1	−6.5	10.6		
Change from baseline to 6 months		15.1	6.5	23.7		9.6	1.0	18.2		
Change from baseline to 12 months		2.1	−6.4	10.7		6.4	−2.2	15.0	0.0006		0.04

Results are presented as mean values, and their 95% CI are based on the mixed models.

**FIGURE 4:**
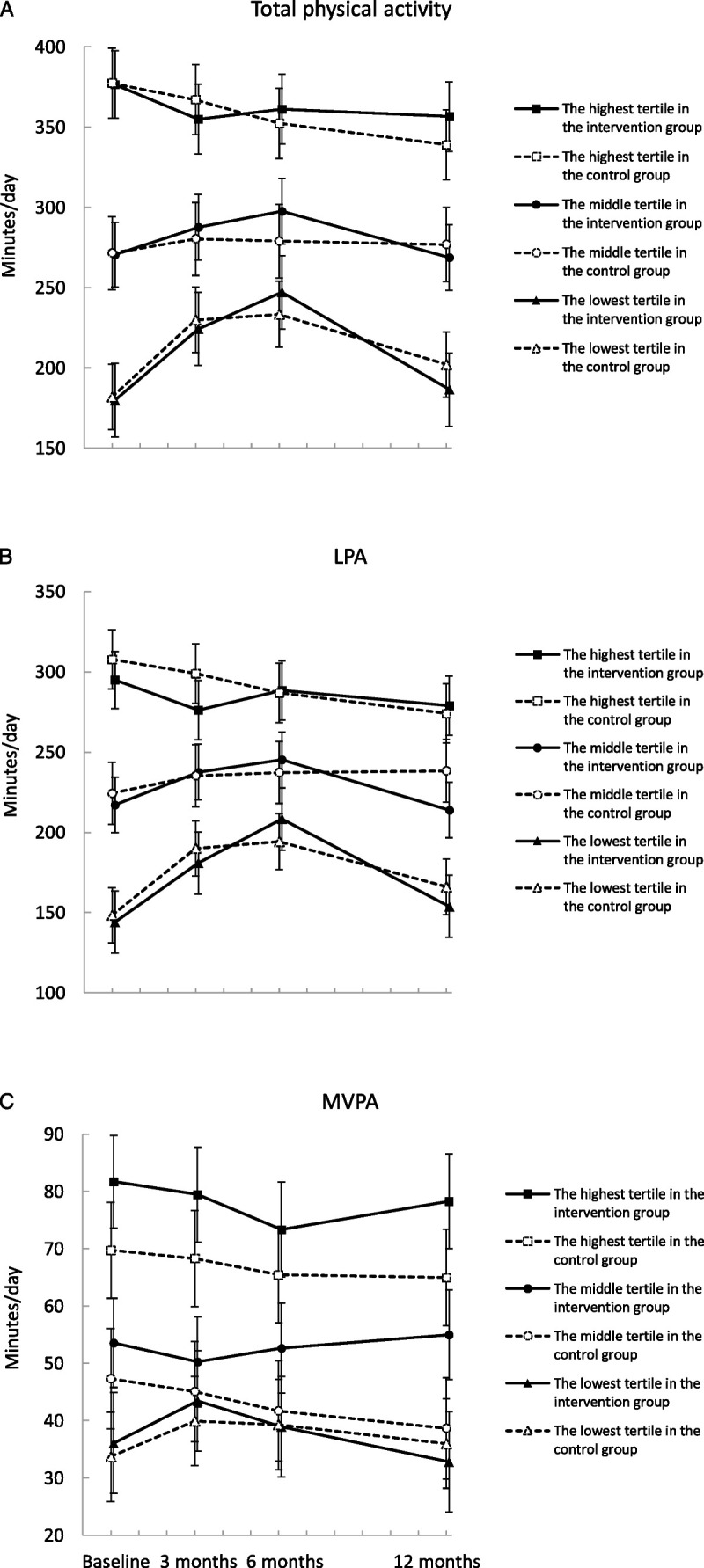
The change in total physical activity (A), LPA (B), and MVPA (C) for the members in the intervention (*solid line*) and control (*dotted line*) groups by the baseline activity tertiles. Results are expressed as mean values and 95% CI based on mixed models.

There were no differences in the changes in total, LPA, and MVPA between the groups by the baseline activity tertiles over the 12 months (tertile–group–time interaction *P* value = 0.54, 0.33, and 0.27, respectively). The stratified analysis showed an increase in total physical activity among the lowest activity tertile over the first 6 months: 67 min (95% CI = 42 to 93) for the intervention and 52 min (95% CI = 29 to 74) for the control group, but no difference was observed between the groups (16 min·d^−1^, 95% CI = −18 to 50) (Fig. 4). Similarly, the intervention participants in the lowest activity tertile increased daily LPA from baseline to 6 months by 64 min (95% CI = 41 to 86) and the control participants in the lowest activity tertile by 46 min (95% CI = 26 to 66), but no difference was observed between the groups (18 min·d^−1^, 95% CI = −12 to 48). The total and the LPA levels returned to a lower level in both groups at 12 months (Fig. 4).

## DISCUSSION

The REACT trial is the first consumed-based activity tracker intervention targeted for the time immediately after retirement when people have been found to be prone to increase their physical activity (3,5–8). The results from our long-term trial showed that the use of a commercial activity tracker did not increase accelerometer-measured daily total physical activity, LPA, or MVPA over the 12 months among recent retirees, when compared with controls not using the activity trackers. However, there was an increase in the LPA over the first 6 months, especially among the participants in the lowest baseline activity tertile, but the change was not significantly different from that of the controls, and the levels lowered near to baseline levels in both groups at the 12-month end point.

The commercial activity tracker used in the REACT trial included self-management strategies, such as goal setting, self-monitoring, and feedback, which have previously been identified as features of a successful technology-aided intervention among older adults (21,43) and which are comparable with those of other commercial activity trackers (34). As the purpose of this study was to examine the effect of a low-cost and easily scalable method to promote daily physical activity among recently retired adults, the intervention consisted of the use of the commercial activity tracker, and no other methods were included. Consequently, the behavioral target for the intervention was defined in terms of the daily activity goals inherent in the activity tracker. Although the daily activity goal used in this study is not comparable with the previous intervention studies using 150 min·wk^−1^ of MVPA or 10,000 daily steps as the goal, the daily activity goal at stage 1 already exceeded the recommendations of 150 min·wk^−1^ of MVPA (35).

The results of our study showed that the 12-month use of a commercial activity tracker can induce short-term albeit nonsignificant changes in LPA among recent retirees. The short-term finding is somewhat comparable with previous multicomponent interventions using wearable devices (29–33) and showing an increase in mainly MVPA up to 6 months among older adults. However, in the previous trials, the physical activity goals were set according to instructions or programs on how to achieve the goal (e.g., 150 min of weekly MVPA or 10,000 daily steps). In the REACT trial, the daily activity goal included the possibility to accumulate various activities at different intensities, and no guidance on how to achieve the daily activity goal was given by the researchers. These differences in the intervention aims, durations, and methods between the present tracker-based and the previous multicomponent interventions can explain why we saw no changes in MVPA but some temporary increase in LPA, especially among the least active participants. However, although the short-term finding favored intervention participants, the changes were not significantly different from those of the control retirees not using the trackers. Therefore, integrated methods, such as counseling (31,44,45) or a Web-based intervention (29,32,33,46), may be needed to induce more marked changes in physical activity (27).

It is plausible that the daily activity goals in the present study were too easily achieved by some of the intervention members. Among the highly active participants, the activity tracker could have only been used as a monitoring equipment, and more features (e.g., heart rate) might have been needed to increase physical activity (27,45,47). Furthermore, our long-term findings, among others (25,27), showed that the changes in physical activity elicited by the activity trackers may only be temporary. In fact, the overall usage of the wearable devices has been shown to be rather short-term among older adults (47). Therefore, more long-term interventions are needed to examine which integrated or alternative methods are effective in producing long-term changes in physical activity habits in this age group.

### 

#### Strengths and limitations

The REACT trial has several strengths. The participants were recruited according to their actual dates of retirement, which enabled us to target the intervention to a time window immediately after retirement. The REACT trial included accelerometer-based outcome measurements at four different follow-up time points over 12 months so as to capture both short- and long-term changes. The adherence to the outcome measurements among both intervention and control group members was excellent as the dropout rate was only 2%. All analyses were performed by intention-to-treat principle. In addition, we were able to follow and control the individual usage of the trackers and to evaluate the dose of activity among the intervention participants.

There are also limitations that should be acknowledged. First, the wrist-worn accelerometers were selected to measure the whole 24-h behavior, but they are not accurate to detect, e.g., cycling (48). Therefore, we may not have been able to measure all modes of daily activities. Second, the baseline measurements (e.g., body composition) may have motivated participants to change their physical activity behavior irrespective of their group allocation. Also, the “wear effect” from the wrist-worn accelerometer devices during the follow-up measurements may have increased physical activity levels among the control group participants although the accelerometers did not give any feedback to the users. Third, the retirees who took part in the study were rather healthy and active older adults. The high proportions of women and highly educated adults in the study are in agreement with the characteristics of public sector employees in Nordic welfare state settings, but they may limit the generalizability of our findings. Finally, it remains to be investigated whether increases in total physical activity, although nonsignificant but still some 24 min·d^−1^, are translated to better health marker outcomes when compared with the participants’ own baseline or the control group.

#### Conclusions

The use of a commercial activity tracker does not elicit significant changes in daily total physical activity over 12 months among a general population sample of recent retirees. Although the activity trackers may be feasible in supporting physical activity engagement (27), the findings of this study highlight the need to explore other alternatives or complementary strategies to increase physical activity after retirement.

## Supplementary Material

SUPPLEMENTARY MATERIAL
