# Supplementary material for: The Effect of Consumer-based Activity Tracker Intervention on Physical Activity among Recent Retirees—An RCT Study
Source: Med Sci Sports Exerc. 2021 Feb 8;53(8):1756–65. doi: 10.1249/MSS.0000000000002627 (PMC8284385; doi:10.1249/MSS.0000000000002627)
Supplement: SUPPLEMENTARY MATERIAL [file msse-53-1756-s001.docx]

**Supplementary file 1. Intervention content described in terms of behavior change techniques (BCTTv1).**

| **[#] Behavior Change Technique (BCTTv1)** | **Mode of Delivery** | |
| --- | --- | --- |
|  | **Activity Tracker**  **(Polar Loop 2)** | **Web-based Software/Application**  **(Polar Flow)**** |
| [12.5] Adding objects to the environment | The intervention group were given activity trackers and requested to wear them every day and night for 12 months. |  |
| [1.1] Goal Setting (behavior) | Participants in the intervention group were instructed to try to reach the daily activity goal, initially set at Stage 1 according to the goals set by the tracker manufacturer. The daily activity goals were based on user’s typical daily activities, and they were sensitive to the user’s gender and age.  The manufacturer’s web-based program/application recommended Stage 1 for individuals whose days included only a little physical activity and a lot of sitting, Stage 2 for individuals who spent most of their days on their feet due to the type of work or daily chores, and, Stage 3 for individuals whose work was physically demanding or who tended to be on the move and highly physically active.  Examples of the amount of physical activity necessary for meeting the set goals:  Stage 1: 57min of jogging OR 2h 11min of walking OR 7h 20min of household chores OR a combination of activities at different intensities.  Stage 2: 1h 13min of jogging OR 2h 47min of walking OR 9h 20min of household activities OR a combination of activities at different intensities.  Stage 3: 1h 29min of jogging OR 3h 23min of walking OR 11h 20min of household chores OR a combination of activities at different intensities. |  |
| [1.5] Review behavioral goals | If a participant frequently exceeded the daily activity goal, a higher goal was suggested by the researcher via e-mail. |  |
| [1.6] Discrepancy between current behavior and goal | Based on the current level of attained daily activity, the activity tracker displayed how much activity is still required for reaching the daily goal (e.g., “To go: jog for 20 minutes or walk for 50 minutes or up for 2h 30 minutes”). |  |
| [2.3] Self-monitoring of behavior | The activity tracker enabled the participant to monitor the accumulation of daily activity and, e.g., daily steps in real-time.***** | Personal accounts were created by the researcher in Polar Flow where the participants were instructed to upload data from their trackers at least once a week. By presenting overviews and summaries on a daily, weekly and monthly level, Polar Flow allowed for long-term monitoring of the data collected by the tracker (activity, sedentariness, sleep). The participants had unrestricted access to their Polar Flow accounts and, upon uploading the data from the activity tracker, the diary view of the Polar Flow was opened in the web browser (see Supplementary file 2). |
| [2.2] Feedback on behavior | The activity tracker displayed real-time feedback on the level of attainment of the daily activity goal. | Polar Flow presented participants with feedback on daily activity goal attainment on a daily, weekly and monthly basis. If the activity tracker had been worn sufficiently, Polar Flow also provided detailed verbal feedback on accumulated activity and sedentariness and their consequences for health.  Requirements for wear time for detailed feedback:  10h per day (for daily feedback)  On at least 5 days of the week (for weekly feedback)  On at least 21 days of the month (for monthly feedback) |
| [7.1] Prompts/Cues | After 55min of uninterrupted sedentary time, the activity tracker gave an inactivity alert by vibrating and showing a prompt “It’s time to move” on the display. | If the participant failed to respond to the inactivity alert within 5 minutes, an “inactivity stamp” was recorded in Polar Flow and presented in the diary view ([2.3] Self-monitoring of behavior). |
| [10.4] Social rewards | The activity tracker informed and congratulated the user upon successful attainment of the daily activity goal. | If worn sufficiently, Polar Flow provided verbal feedback and praise on accumulated activity on a daily, weekly, or monthly level. For wear time requirements, see above ([2.2] Feedback on behavior). |
| [5.1] Information about health consequences |  | If worn sufficiently, Polar Flow presented detailed information about health consequences of accumulated activity. For wear time requirements, see above ([2.2] Feedback on behavior). |

*The activity tracker also displayed daily consumption of calories if the user had provided height and weight information in Polar Flow.

**Polar Flow contained several additional functionalities, such as provision of training programs, possibilities for using various social media utilities, recording of behavioral and emotional outcomes, and provision of monthly reports of the data collected by the tracker (including a list of “best days” on various measures). However, as the participants were not instructed to use nor informed about these functionalities, they were not coded here as active intervention components.
