# Supplementary material for: The Effect of Consumer-based Activity Tracker Intervention on Physical Activity among Recent Retirees—An RCT Study
Source: Med Sci Sports Exerc. 2021 Feb 8;53(8):1756–65. doi: 10.1249/MSS.0000000000002627 (PMC8284385; doi:10.1249/MSS.0000000000002627)

**Supplementary file 2. Polar Flow diary view (no patient data, published with permission from Polar).**


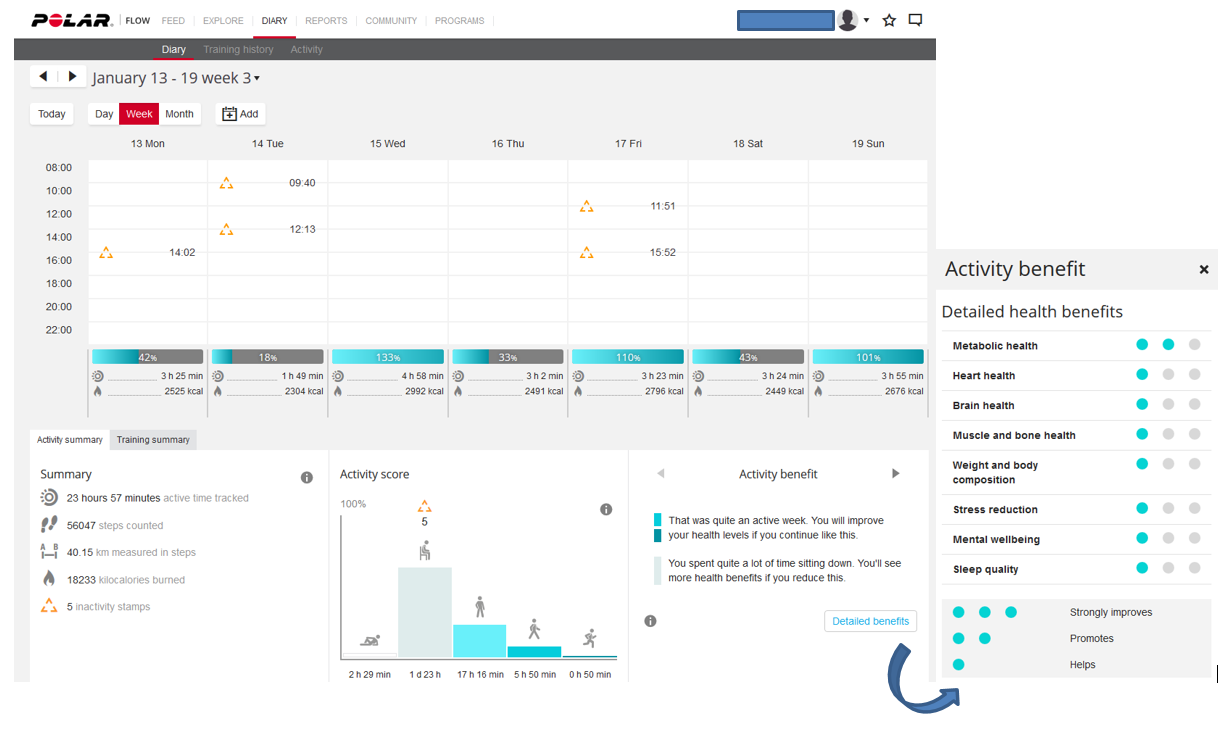

Supplement: SUPPLEMENTARY MATERIAL [file msse-53-1756-s002.docx]
