# Supplementary material for: The Effect of Consumer-based Activity Tracker Intervention on Physical Activity among Recent Retirees—An RCT Study
Source: Med Sci Sports Exerc. 2021 Feb 8;53(8):1756–65. doi: 10.1249/MSS.0000000000002627 (PMC8284385; doi:10.1249/MSS.0000000000002627)
Supplement: SUPPLEMENTARY MATERIAL [file msse-53-1756-s003.docx]

**Supplementary file 3. Active time (min/day) per each intervention month from the Polar Loop 2 activity tracker data.** Values are expressed as means and 95% CIs based on mixed models.

Participants were treated in five waves starting the 12-month intervention at spring season (44% of the participants), autumn season (25%) and winter season (31%). Overall, 55% of the participants kept the initial daily activity goal throughout the intervention.
